# Care participation and burden among informal caregivers of older adults with care needs and associations with dementia

**DOI:** 10.1017/S104161021500160X

**Published:** 2015-10-19

**Authors:** Janhavi Ajit Vaingankar, Siow Ann Chong, Edimansyah Abdin, Louisa Picco, Anitha Jeyagurunathan, YunJue Zhang, Rajeswari Sambasivam, Boon Yiang Chua, Li Ling Ng, Martin Prince, Mythily Subramaniam

**Affiliations:** 1 Research Division, Institute of Mental Health, 10 Buangkok View, Singapore; 2 Changi General Hospital, 2 Simei Street 3, Singapore; 3 King's College London, Strand, London, UK

**Keywords:** behavioral and psychological symptoms of dementia, care need, correlates of care burden, Zarit Burden Interview

## Abstract

**Background:**

Few studies have estimated care burden in large, representative, multi-ethnic Asian population-based informal caregivers of older adults with care needs. This study describes informal caregivers’ care participation for a population-based sample of older adults with care needs in Singapore, investigates differences by dementia status, and examines correlates of caregivers’ burden.

**Methods:**

Data collected from 693 pairs of older adults, aged 60 to 100 years, having any care needs, and their informal caregivers, who were aged 21 to 88 years, closely involved in their care and “knew the older resident best,” and were interviewed during a cross-sectional national survey, were used. Clinical characteristics of older adults, including behavioral and psychological symptoms of dementia (BPSD) and dementia diagnosis, care needs, and socio-demographic characteristics of participants were obtained. Care burden was assessed with the Zarit Burden Interview.

**Results:**

Informal caregivers’ participation was highest in activities related to communication (35.1%), feeding (32%), and bathing (21.1%). Among the older adults with any care need, 356 (51.4%) had dementia. Care burden was significantly associated with married caregivers (odds ratio (OR) 2.4 vs. never married), when their relative belonged to a younger cohort (OR 2.5 vs. >84 years), needed care much of the time (OR 2.5 vs. no care needed), exhibited BPSD (OR 3.5 vs. no BPSD), and had dementia (OR 2.52 vs. no dementia).

**Conclusions:**

Factors related to older adults – more care needs, presence of BPSD, and dementia – were significant contributors to informal caregivers’ burden, and these should be considered while planning interventions to alleviate care burden.

## Introduction

Older populations typically have multiple medical problems, huge healthcare utilization, require multiple service approaches, and need caregivers to take care of their health and care requirements (Bähler *et al.*, 2015; Huang *et al.*, 2015), all of which impose significant challenges in their management. With the focus on rehabilitation and managing care in the right setting, such as the community, the role and process of caring for older adults often emerges naturally among families. While benefits of active informal caregiving result in better patient management and lower resource utilization for the healthcare systems, the personal cost to the family caregiver can be substantial. As a result, informal caregivers of older adults often experience significant long-term burden of care (Prince *et al.*, 2012; Ikeda *et al.*, 2015). Informal caregivers’ role varies by the care need of older adults, which often precedes a formal diagnosis of common geriatric illnesses such as stroke or dementia (Prince *et al.*, 2004), and these influence the care burden experienced by caregivers. However, extant findings on the relationship of caregivers’ socio-demographic characteristics, such as age, gender, and the relationship with the older adult, on care burden are inconclusive (Dunkin and Anderson-Hanley, 1998; Gallicchio *et al.*, 2002; Chumbler *et al.*, 2003).

Singapore is a developed sovereign city-state in Southeast Asia, situated off the southern tip of the Malay Peninsula. In 2013, the country's resident population (Singapore Citizens and Permanent Residents) stood at 3.8 million, comprising Chinese (74.2%), Malay (13.2%), Indian (9.2%), and other ethnicities (3.3%) (http://www.singstat.gov.sg). With advances in healthcare and population well-being, the life expectancy rate among those aged 65 years and above has increased from about 17 years in 2000 to 20.2 years in 2013 (http://www.singstat.gov.sg). However, with the faltering family size and fertility rate, the old-age support ratio of residents has continued to fall from 13.5 in 1970 to 8.4 in 2000 to 6.4 in 2013. While the older adult population is expected to triple to 900,000 by 2030, there will only be 2.1 working-age citizens for each citizen aged 65 years and above (http://app.msf.gov.sg/portals/0/summary/research/Materials_IMCReport.pdf). This presents several challenges in terms of care and support, and highlights the critical role of informal caregivers in elder care in Singapore.

Although caregivers’ burden and its predictors have been extensively studied among clinical and help-seeking older populations (Etters *et al.*, 2008), there has been insufficient research on nationally representative samples in developed nations and particularly in multi-ethnic Asian populations. Thus, there is still a need to identify the major factors contributing to caregivers’ burden in Asian samples based on large-scale representative data using appropriate methodology among general caregiver populations. Previous studies among Singapore's older population have reported a correlation between dementia and caregivers’ burden (Mehta, 2005; Tan *et al.*, 2005; Tew *et al.*, 2010). However, these studies were limited in terms of the sample size and focused on specific population groups. Moreover, with the recent changes in caregiver demographics, healthcare policies, and resulting impact on care arrangements for older residents, it is of interest to establish the socio-demographic profiles of their informal care providers and investigate the presence and correlates of care burden among family caregivers in a large population sample.

Data for this study were collected during the Well-being of the Singapore Elderly (WiSE) study, which was a large, population-based, cross-sectional survey conducted to estimate the prevalence and factors associated with dementia in Singapore. The present study aimed to (1) describe the care participation, care needs, and care burden among informal caregivers of a cohort of older adults with care needs in Singapore, (2) investigate the association of dementia with these, and (3) examine factors related to the care burden experienced by their informal caregivers.

## Methods

The WiSE survey was conducted across Singapore with older adults aged 60 years and above and their respective family member or a close friend – referred to as “informant.” Ethical approval to conduct the study was obtained from the relevant ethics committees. Study details have been explained elsewhere (Subramaniam *et al.*, 2015).

Briefly, older adults aged 60 years and above were randomly selected from a national database with information on all residents in Singapore. Those who were younger than 60 years, without any address or with overseas addresses as of June 2011, and all foreigners were excluded from the frame. A probability sample was randomly selected using a disproportionate stratified sampling design with 12 strata defined according to ethnicity (Chinese (38.5%), Malay (30%), Indian (30%), others (1.5%)) and age groups (60 to 74, 75 to 84, and 85 years and above). Residents aged 75 years and above, Malays, and Indians were over-sampled to ensure that sufficient sample size would be achieved to improve the reliability of estimates for the subgroup analyses and to account for cultural differences in the three main ethnic groups in Singapore. This study involved a single-stage design without geographic clustering. A total of 2,565 older adults were interviewed between August 2012 and November 2013, giving a response rate of 66%.

Informed consent was obtained from participants themselves or through their legally acceptable representatives (for those participants who were mentally or physically incapable to consent themselves). Each older participant was then asked to identify one family member or friend, who according to the older participant was most involved in their care and “who knew the older adult best,” and therefore best suited to participate in the survey. Informants were eligible for the survey if they were Singapore residents, aged 21 years and above, and were able to provide adequate and accurate information on the older person's health condition and service use. While some informants were caregivers (i.e. actively involved in care provision of the older person), others were co-residents or other close contacts with no caregiving role. Approximate time spent with the older person was used as a criterion for deciding the best informant in the situation where there were several co-resident family members involved in the care or decision-making of the older person. Informants were excluded from the sample if (a) interviewers were unable to contact them after ten contact attempts, (b) they were non-residents of Singapore, residing outside Singapore at the time of the survey, (c) they were unwilling or unable to complete the interview in any of the four languages – English, Chinese (either Mandarin or one of three dialects – Hokkien, Teochew, or Cantonese), Malay, or Tamil, or (d) they were paid caregivers.

A total of 2,421 informants were interviewed during the survey; 117 of the 2,565 older adults did not have any relative or friend who could be interviewed, or refused to refer another family member for the survey, or potential informal caregivers were ineligible based on resident status, age, language, or mental/physical capacity. Among the remaining, 22 refused to participate in the survey and five could not be contacted or interviewed after repeated attempts, resulting in an overall loss of information in about 5% of the sample of informants. For this paper, data from 693 (28.6% of the full survey sample) pairs of older adults who needed any care and their informants, who were also their informal caregivers, were used. “Informants” of this group of older adults are referred to as “informal caregivers,” henceforth.

The 10/66 Study Protocol was adapted for this study (Prince *et al.*, 2007). Details are provided in another article (Subramaniam *et al.*, 2015). A 10/66 dementia diagnosis was obtained with the Geriatric Mental State (GMS) using a computerized algorithm (AGECAT) along with other measures such as the Community Screening Interview for Dementia (CSI-D), Consortium to Establish a Registry for Alzheimer's Disease (CERAD) test battery, History and Aetiology Schedule – Dementia Diagnosis and Subtype (HAS-DDS), and neurological assessments (Prince *et al.*, 2007). The 10/66 dementia diagnosis, established to define a “broader category of dementia that may be more sensitive, identifying genuine cases beyond those defined by the Diagnostic and Statistical Manual, Fourth Edition (DSM-IV) algorithm, with relevance to the estimation of the population burden of this disorder,” has been validated against the DSM-IV diagnoses (Prince *et al.*, 2008; Phung *et al.*, 2014; Subramaniam *et al.*, 2015).

The following information was collected from the informal caregivers.

*Socio-demographic information*: Information on older adults’ age and gender and caregivers’ age, gender, ethnicity, marital status, education, relationship to the older adult, whether they lived with the older person, etc. were collected.

**Figure 1.**
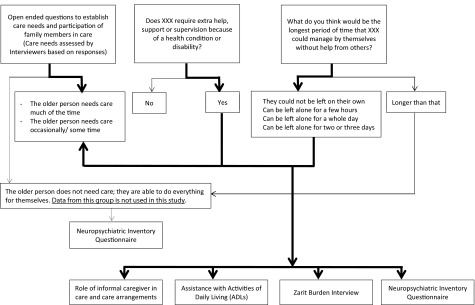
Flow diagram of data collection from informal caregivers on care needs of older adults.

*Care need, care arrangement, and care participation of informal caregivers*: Data on informants’ involvement in the care of an older adult were collected as shown in Figure 1. They were asked to report if the older adult “required extra help, support, or supervision because of a health condition or disability” and what was the “longest period of time (a few hours/whole day/two to three days/longer than that) they could manage by themselves, without help from others, supposing they were living on their own.” Interviewers also collected information on care arrangements using supplemental open-ended questions from all informants (who lives with X?, what kind of help is needed by X inside and outside the house?, who provides this help?, etc.). Informants who reported “No” to the first question and “longer than that” to the second, did not answer questions related to the care needs. Based on this, and responses to the open-ended questions, the interviewers then assessed whether the older adult needed care “much of the time, some of the time, or did not need care” (i.e. they were able to do everything for themselves). Informants of older persons who “did not need care” were not asked further questions related to their care needs and their data were excluded from this study. Informal caregivers of older adults with some/much care needs were then asked questions on their involvement in care for the older person using a list of eight activities of daily living (ADLs) – communicating with the person, supervising the person, and helping them with using transport, dressing, eating, grooming (looking after their appearance), toileting, and bathing. These activities were presented to the caregivers, and they were asked to report “how much time they spent doing each of these in the past 24 h.” Information on their participation in each was captured using four options – no time, less than 1 h, 1 to 2 h, more than 2 h. Information on care arrangements, such as whether the caregiver was the “main hands-on” or “organizational” caregiver, whether they had given up or cut down on their work to care for the older person and the involvement of other family members/friends/paid-helpers, etc were also captured.

*Behavioral and psychological symptoms of dementia (BPSD) and care burden*: The Neuropsychiatric Inventory Questionnaire (NPI-Q) was used to assess the presence of BPSD during last month by the older adult on the following 12 categories: delusions, hallucinations, anxiety, depression/dysphoria, agitation/aggression, elation/euphoria, disinhibition, irritability/lability, apathy/indifference, motor disturbance, night-time behavior problems, and problems with appetite/eating (Kaufer *et al.*, 2000). Informal caregivers were asked whether the older person exhibited each of these behaviors (e.g. “Does X believe that others are stealing from him or her, or planning to harm him or her in some way?,” “Does X act as if he/she is sad or in low spirits?”).

Informal caregivers were also administered the Zarit Burden Inventory (ZBI; Zarit *et al.*, 1980), which is a widely used 22-item self-report inventory that measures subjective care burden among caregivers. Its validity has been established in Singapore (Seng *et al.*, 2010). The scale items examined burden associated with functional/behavioral impairments and the home care situations. Each item was scored on a 5-point Likert Scale ranging from “never” to “nearly always present.” A total score was obtained, with higher scores indicting higher care burden among informal caregivers. The ZBI scores were dichotomized using a cut-off of 24 (Schreiner *et al.*, 2006) to identify correlates of care burden in this group.

### Statistical analysis

Descriptive statistics for socio-demographic variables and measures applied to caregivers were computed using the SPSS version 18.0. *χ*^2^-tests and Mann–Whitney U tests were used for comparisons of categorical and continuous variables respectively among informal caregivers of those with and without dementia. To identify factors related to care burden, multivariate logistic regression analyses were performed with the presence of burden being the dependent variable, and older adults’ age, gender, care need, presence of BPSD, and dementia status, and caregivers’ age, gender, ethnicity, marital status, education level, having children, relationship with the older adult, whether they lived with the older person, and, received paid or unpaid help with care, as independent variables. Two-sided tests were applied and statistical significance was set at *p* < 0.05.

## Results

### Socio-demographic profile of informal caregivers of older adults with care need

The mean age of the informal caregivers was 54.5 (SD 12.9) years, ranging from 21 to 88 years. The majority of the caregivers were aged between 50 and 64 years (45.1%), were women (66.0%), with secondary education (41.8%), and married (65.5%) (Table 1).

**Table 1.** Socio-demographic profile of informants of older residents

				informal	
				caregivers of	
				older adults	
		sample		with any care	
		(*N* = 2,421)		need (*n* = 693)	
		*n*	%	*n*	%
Age group (years)	21–34	284	11.7	46	6.6
	35–49	588	24.3	186	26.9
	50–64	908	37.6	312	45.1
	65+	638	26.4	148	21.4
Gender	Women	1,559	64.4	457	66.0
	Men	861	35.6	235	34.0
Ethnicity	Chinese	931	38.5	230	33.2
	Malay	728	30.1	250	36.1
	Indian	728	30.1	208	30.0
	Others	34	1.4	5	0.7
Marital status	Never married	528	21.8	180	26.1
	Married/cohabiting	1,720	71.2	452	65.5
	Separated/divorced	116	4.8	37	5.4
	Widowed	53	2.2	21	3.0
Having children	No	686	28.4	224	32.5
	Yes	1,731	71.6	466	67.5
Education level	None	80	3.3	18	2.6
	Minimal	213	8.8	51	7.4
	Completed primary	516	21.3	137	19.8
	Completed secondary	870	36.0	289	41.8
	Completed tertiary	740	30.6	196	28.4
Relationship with the older resident	Spouse	883	36.5	131	18.9
	Son/daughter	1,082	44.7	428	61.8
	Son/daughter-in-law	147	6.1	71	10.3
	Sibling	97	4	16	2.3
	Other relative	108	4.5	33	4.8
	Friend/neighbor or other	103	4.3	13	1.9
Lives with the older resident	No	425	17.6	125	18.1
	Yes	1,994	82.4	567	81.9

### Care need, care arrangement, and care participation of informal caregivers

Out of the 693 older adults with any care need, 330 (47.6%) needed care “much of the time” and 363 (52.4%) needed care “some of the time” (Table 2). Among the older adults with any care need, 356 (51.4%) had dementia. Care needs and care arrangements reported by informal caregivers varied for those without and with dementia. Over 80% of older adults with dementia needed care some or much of the time. Significantly, higher proportion (64.3%) of those with dementia needed care much of the time as compared with 30% of those without dementia. Assistance received by caregivers from other family members or friends was similar in the two groups; however, a significantly higher proportion of informal caregivers of older people with dementia received help from a paid helper (Table 2). The top three care roles undertaken by caregivers of older adults with any care need were communicating with the person (13.5%), supervising the older adult (8.6%), and feeding (3.6%). Except for assistance with transport, statistically significant and higher proportion of caregivers of people with dementia spent time on care tasks, with the highest being for feeding (Table 3).

**Table 2.** Care needs and care arrangements reported by informal caregivers (*N* = 693)

				without		with		
		overall		dementia		dementia		
		*n*	%	*n*	%	*n*	%	*p*-value
Care needs	Needs care much of the time	330	47.6	101	30.0	229	64.3	**<0.001**
	Needs care some of the time	363	52.4	236	70.0	127	37.7	
Informal caregivers’ involvement in care	One of the main “hands on” caregivers	396	57.1	200	59.3	196	55.1	**<0.001**
	One of the main “organizational” caregivers	223	32.2	84	24.9	139	39.0	
	Only slightly involved in providing or organizing care	65	9.4	47	13.9	18	5.1	
	Not at all involved in providing or organizing care	9	1.3	6	1.8	3	0.8	
Who provides “hands on’ care	One or more family members	442	64.8	243	73.0	199	57.0	**<0.001**
	One or more unpaid friends or neighbors	8	1.2	7	2.1	1	0.3	
	One or more paid caregivers	232	34	83	24.9	149	42.7	
Given up or cut down on work	Yes, given up	55	8.1	22	6.7	33	9.4	0.301
	Yes, cut down	76	11.2	34	10.3	42	12.0	
	No	550	80.8	274	83.0	276	78.6	
Friends or relatives help with care	No	559	80.8	277	82.2	282	79.4	0.357
	Yes	133	19.2	60	17.8	73	20.6	
Paid help during the day	No paid help	384	55.7	223	66.8	161	45.4	**<0.001**
	Occasional	14	2	6	1.8	8	2.3	
	Regular	98	14.2	37	11.1	61	17.2	
	Constant	193	28	68	20.4	125	35.2	
Paid help during the night	No paid help	503	73.5	266	80.1	237	67.3	**<0.001**
	Sleep-in paid help	141	20.6	49	14.8	92	26.1	
	Waking paid help	40	5.8	17	5.1	23	6.5

**Table 3.** Informal caregivers’ participation in activities of daily living (ADLs) of older residents (*N* = 693)

				without		with		
		overall		dementia		dementia		
		
	time spent in							
	24 hours	*n*	%	*n*	%	*n*	%	*p*-value
Communicating	No time	401	59.1	235	71.4	166	47.4	**<0.001**
with the person	1 to 2 h	186	27.4	69	21.0	117	33.4	
	More than 2 h	92	13.5	25	7.6	67	19.1	
Using transport	No time	599	87.4	288	86.0	311	88.9	0.708
	Less than 1 h	40	5.8	22	6.6	18	5.1	
	1 to 2 h	23	3.4	13	3.9	10	2.9	
	More than 2 h	23	3.4	12	3.6	11	3.1	
Dressing	No time	527	76.6	292	87.4	235	66.4	**<0.001**
	Less than 1 h	130	18.9	34	10.2	96	27.1	
	1 to 2 h	21	3.1	5	1.5	16	4.5	
	More than 2 h	10	1.5	3	0.9	7	2.0	
Feeding	No time	445	64.6	258	77.0	187	52.8	**<0.001**
	Less than 1 h	172	25	60	17.9	112	31.6	
	1 to 2 h	47	6.8	10	3.0	37	10.5	
	More than 2 h	25	3.6	7	2.1	18	5.1	
Grooming	No time	563	81.8	306	91.6	257	72.6	**<0.001**
	Less than 1 h	95	13.8	22	6.6	73	20.6	
	1 to 2 h	18	2.6	3	0.9	15	4.2	
	More than 2 h	12	1.7	3	0.9	9	2.5	
Supervising the person	No time	542	80.5	289	87.8	253	73.5	**<0.001**
	1 to 2 h	73	10.8	23	7.0	50	14.5	
	More than 2 h	58	8.6	17	5.2	41	11.9	
Helping with toileting	No time	542	78.8	296	88.6	246	69.5	**<0.001**
	Less than 1 h	98	14.2	26	7.8	72	20.3	
	1 to 2 h	36	5.2	9	2.7	27	7.6	
	More than 2 h	12	1.7	3	0.9	9	2.5	
Helping with bathing	No time	526	76.2	291	86.9	235	66.2	**<0.001**
	Less than 1 hour	124	18	38	11.3	86	24.2	
	1 to 2 h	30	4.3	3	0.9	27	7.6	
	More than 2 h	10	1.4	3	0.9	7	2.0	

### BPSD and care burden

At least one BPSD was present among 381 (55.2%) of the older adults with care needs with a significantly higher proportion (*p* < 0.001) among those with dementia (66.1%) versus those without (33.9%). The mean ZBI care burden score among the informal caregivers was 14.0 ± 13.0 (range: 0–88). ZBI scores among caregivers of people without (10.8 ± 10.6) and with dementia (17.0 ± 14.2) differed significantly (*p* < 0.001). Among the informal caregivers, 24.5% met criterion for care burden.

### Correlates of care burden among informal caregivers

The correlates of care burden examined using logistic regression analyses are reported in Table 4. Caregivers with children had 0.4 times lower risk of burden than those without children. Care burden was significantly associated with caregivers’ marital status: compared with those who had never married, married caregivers had a 2.4 times higher risk of burden. Informal caregivers were also more likely to experience care burden if their relative belonged to a younger cohort (2.5 times higher risk among residents aged 60–74 years than those aged 84 years and above), needed care much of the time (2.5 times), exhibited BPSD (3.5 times), and had dementia (2.5 times).

**Table 4.** Correlates of care burden assessed by ZBI among informal caregivers of older residents having some or much care need (*N* = 693)

		or	95% CI		*p*-value
Caregiver's characteristics					
Age (years)	21–34	Ref.			
	35–49	2.6	0.8	8.1	0.099
	50–64	2.3	0.7	7.2	0.168
	65+	1.7	0.5	6.8	0.422
Gender	Women	1.1	0.7	1.7	0.703
	Men	Ref.			
Ethnicity	Chinese	Ref.			
	Malay	0.6	0.4	1.0	0.071
	Indian	1.2	0.7	2.1	0.458
	Others	1.8	0.1	21.8	0.657
Marital status	Never married	Ref.			
	Married/cohabiting	2.4	1.1	5.5	0.034
	Separated/divorced	1.5	0.5	4.5	0.432
	Widowed	2.2	0.5	8.9	0.27
Having children	No	Ref.			
	Yes	0.4	0.2	0.8	0.006
Education	None	1.3	0.3	5.2	0.697
	Minimal	1.0	0.4	2.6	0.93
	Completed primary	0.8	0.4	1.6	0.567
	Completed secondary	1.1	0.6	1.8	0.798
	Completed tertiary/further education	Ref.			
Relationship with the older person	Spouse	Ref.			
	Child	1.4	0.6	3.5	0.448
	Other relative/friend	0.8	0.3	2.0	0.563
Lives with the older person	No	Ref.			
	Yes	1.2	0.7	2.1	0.604
Receives help with care	No	Ref.			
	Yes	1.3	0.8	2.1	0.225
Older residents’ characteristics					
Age (years)	60–74	2.5	1.3	4.9	0.006
	75–84	1.4	0.8	2.3	0.256
	85+	Ref.			
Gender	Women	0.9	0.6	1.5	0.705
	Men	Ref.			
Care need	Some	Ref.			
	Much	2.5	1.6	4.0	<0.001
BPSD	None	Ref.			
	At least one	3.5	2.2	5.6	<0.001
Dementia	No	Ref.			
	Yes	2.5	1.5	4.2	0.001

Note: BPSD = Behavioural and psychological symptoms of dementia.

## Discussion

Consistent with the findings from previous research (Prince *et al.*, 2004; Huang *et al.*, 2015; Stoltz *et al.*, 2015), caregivers in our sample participated in various care and related activities for older adults. As expected, care need, assistance with ADLs, and presence of BPSD were significantly higher among older adults with dementia, resulting in higher care participation. For the majority of older adults (65%), hands-on care was provided by one or more of their family members, and for about 20% of them, friends or other relatives also assisted with care. Compared with other studies that have found a proportion ranging from 17–40% (Prince *et al.*, 2004; Brodaty and Donkin, 2009), we had a slightly smaller proportion of caregivers (19.3% of the overall sample and 21.4% of those caring for a person with dementia) who had cut down or given up their work. Regardless, the combined effect of work, need for assistance from other unpaid caregivers, and receipt of help from paid helpers highlight substantial strain among informal caregivers in Singapore, and more so for those caring for an older relative with dementia.

A sizeable proportion of caregivers experienced burden, and this is consistent with other studies (Brodaty and Donkin, 2009; Tew *et al.*, 2010; Prince *et al.*, 2012). The ZBI score among caregivers of people with dementia in our sample was much lower than that reported elsewhere (Hori *et al.*, 2011; Prince *et al.*, 2012; Brodaty *et al.*, 2013). Socio-cultural differences and caregiver characteristics could be possible explanations for this. Lower levels of burden have been reported among Asian and collectivist samples as compared with Western or individualistic populations, which were due to the varying subjective appraisals of care burden in the two cultures and the strong tradition of filial piety among the collectivist societies (Kim *et al.*, 2002; Lai, 2007). Another observation from our study was that for the majority of informal caregivers, there was another member of the family or a paid caregiver to provide help and support (Table 2), and it is possible that the availability of another helper might have resulted in alleviating or lowering the level of burden in our sample, compared with other studies. Lower burden among family members with paid caregivers has been reported from Latin America (Prince *et al.*, 2012) and Japan (Kumamoto *et al.*, 2006).

Several studies that have examined the association between caregiver characteristics and care burden found that kinship ties (spouse, child, and siblings) were an early factor that influence burden among caregivers of older adults, including those with dementia (Almberg *et al.*, 1980; de Vugt *et al.*, 2006). It has been shown that spouses often manifest greater distress and feelings of burden when they alone assume the care of an older individual at home (Vugt *et al.*, 2006). Surprisingly, while we did not observe an association of spousal relationship with care burden in our sample, marital status of caregivers was a strong factor, with higher burden being reported among married caregivers. This finding might have been influenced by the higher proportion of caregivers who were children over spousal caregivers (36%) in our sample. More research is needed in understanding the independent association of spousal relationship over marital status with care burden.

Another finding was the lack of association of caregivers’ gender and education with burden. While there is substantial evidence in literature that female caregivers experience more stress than male caregivers (Gallicchio *et al.*, 2002; Croog *et al.*, 2006), not all studies report clear-cut gender differences (Annerstedt *et al.*, 2000; Chumbler *et al.*, 2003). It is hypothesized that gender may influence the experience of care burden; compared with female caregivers who used emotion-focused coping strategies, male caregivers, on the other hand, tend to adopt more problem-focused coping strategies, which are found to be most effective in reducing strain on caregivers (Almberg *et al.*, 1997). In contrast with other reports which have shown higher burden among primary caregivers with children, we found an inverse association, with lower risk among those with children. Additional care support received from their children could be one of the reasons; however, we did not capture this data specifically in our study. An earlier study has also posited less care burden when more visits were paid by other relatives, particularly children, to the dementia patient (Zarit *et al.*, 1980).

More caregivers of younger older adults experienced burden than those caring for much older individuals. This is in contrast with a number of studies that have reported higher burden among caregivers of older adults. Miyamoto *et al.* (2002) found that caregivers of mobile patients with dementia reported higher amounts of care burden because of more behavioral disturbances than non-mobile patients. More behavioral disturbances have been reported among younger patients with dementia (Beeri *et al.*, 2002), which could possibly explain our finding.

Consistent with previous studies (Beeri *et al.*, 2002; Sink *et al.*, 2005; Tew *et al.*, 2010; Prince *et al.*, 2012), our study found that the presence of BPSD, care need, and dementia in older adults are significant factors contributing to care burden. The effects of BPSD have been reported to be independent and more prominent factors for care burden than cognitive deficits or dementia (Arango, 2009; Prince *et al.*, 2012). We found an almost four times higher risk for burden (among the caregivers of older adults with BPSD as compared with twice the risk for dementia (odds ratio (OR) 2.5). BPSD have also been associated with higher care needs (Kinoshita, 2008; Jeon *et al.*, 2013), further compounding the strain on caregivers, and thus increasing their burden.

A significant strength of the study is the use of a population-based sample with the ability to include older adults needing care and their caregivers not known to the care system – reflecting a broader picture of the situation among informal caregivers. The study also investigated a wider range of correlates of care burden by including multiple factors related to the caregivers themselves, older adults and their care needs, and clinical characteristics such as BPSD and dementia diagnosis.

However, certain limitations need to be considered while interpreting the study findings. This being a cross-sectional study, temporal and causal relationships could not be established. The definition of informal caregivers used in the study was broad – someone who “knew the older person best,” so that they could provide adequate information on older adults’ health status during the survey. The informal caregivers in our study were therefore not necessarily their primary caregivers and this might have influenced some of the observed associations. In addition, a sizeable proportion of older adults in our sample, in particular those with dementia, were cared for by paid helpers and we did not include paid helpers in our study. Caregivers’ participation in ADLs was assessed by estimating the time they spent in the last 24 h before the interview and this might not have adequately covered care provided on an *ad hoc* basis on other days or on a typical day by them leading to under- or over-reporting of the true time spent. Lastly, we could not examine the effect of total time spent on caring, which is an important mediator of care burden as it was not possible to compute the care time accurately due to the categorical nature of responses to care participation and assistance in ADLs.

## Conclusions

This work contributes to improving knowledge concerning care situations of older adults with care needs and care burden experienced by their informal caregivers. The study used large-scale, population-based data that included a number of factors related to care burden after controlling for relevant covariates that ensure generalizability to a wide range of caregiving situations, and broaden our understanding of caregivers’ experienced burden, specifically for those caring for an older adult with dementia. The study has several important implications. The study provides recent information on the scale of caregivers’ burden after taking into account multiple factors, and highlights the need for its assessment in routine clinical practice. We also identified groups at higher risk – married caregivers and caregivers of older adults with more care needs, BPSD, and dementia; and these should be considered while planning interventions to alleviate care burden. The adoption of caregiver support programs for families of older adults with dementia may be able to impact on these findings and improve the experience of their informal caregivers.

## Conflict of interest

None.

## Description of authors’ roles

Janhavi Ajit Vaingankar designed the study, collected, verified, and analyzed data, and wrote the manuscript. Siow Ann Chong and Edimansyah Abdin assisted in study design, interpreted the data, and provided intellectual inputs on the manuscript. Louisa Picco, Anitha Jeyagurunathan, YunJue Zhang, Rajeswari Sambasivam, and Boon Yiang Chua played an active role in data collection, clean up, refining analysis plan, and drafting the manuscript. Li Ling Ng provided clinical inputs into the findings and edited the manuscript. Martin Prince designed and developed the study questionnaires, provided intellectual inputs into the study design, analysis plan, and interpretation of findings. Mythily Subramaniam supervised the overall study design, provided inputs on the manuscript content, and approved the manuscript version to be published.
